# Amino Acid Transporters and Release of Hydrophobic Amino Acids in the Heterocyst-Forming Cyanobacterium *Anabaena* sp. Strain PCC 7120

**DOI:** 10.3390/life5021282

**Published:** 2015-04-23

**Authors:** Rafael Pernil, Silvia Picossi, Antonia Herrero, Enrique Flores, Vicente Mariscal

**Affiliations:** Instituto de Bioquímica Vegetal y Fotosíntesis, Consejo Superior de Investigaciones Científicas and Universidad de Sevilla, Américo Vespucio 49, E-41092 Seville, Spain; E-Mails: rafapernil@gmail.com (R.P.); silvia.picossi@ibvf.csic.es (S.P.); herrero@ibvf.csic.es (A.H.); vicente.mariscal@ibvf.csic.es (V.M.)

**Keywords:** ABC-type transporters, amino acid diffusion, cyanobacteria, membrane transport

## Abstract

*Anabaena* sp. strain PCC 7120 is a filamentous cyanobacterium that can use inorganic compounds such as nitrate or ammonium as nitrogen sources. In the absence of combined nitrogen, it can fix N_2_ in differentiated cells called heterocysts. *Anabaena* also shows substantial activities of amino acid uptake, and three ABC-type transporters for amino acids have been previously characterized. Seven new loci encoding predicted amino acid transporters were identified in the *Anabaena* genomic sequence and inactivated. Two of them were involved in amino acid uptake. Locus *alr2535-alr2541* encodes the elements of a hydrophobic amino acid ABC-type transporter that is mainly involved in the uptake of glycine. ORF *all0342* encodes a putative transporter from the dicarboxylate/amino acid:cation symporter (DAACS) family whose inactivation resulted in an increased uptake of a broad range of amino acids. An assay to study amino acid release from *Anabaena* filaments to the external medium was set up. Net release of the alanine analogue α-aminoisobutyric acid (AIB) was observed when transport system N-I (a hydrophobic amino acid ABC-type transporter) was engaged in the uptake of a specific substrate. The rate of AIB release was directly proportional to the intracellular AIB concentration, suggesting leakage from the cells by diffusion.

## 1. Introduction

Cyanobacteria are a group of prokaryotic microorganisms characterized by their ability to fix CO_2_ at the expense of oxygenic photosynthesis. They represent a coherent phylogenetic group in spite of showing a very diverse morphology and having colonized many different natural habitats. Some cyanobacteria are true multicellular organisms, growing as chains of cells that communicate between them and divide labor between specialized cells. Under combined nitrogen deprivation, heterocyst-forming cyanobacteria present two cell types: vegetative cells that perform oxygenic photosynthesis and heterocysts that carry out N_2_ fixation [1]. These specialized cells rely on each other: heterocysts require photosynthate that is provided by vegetative cells, and heterocysts provide vegetative cells with fixed nitrogen [2,3].

Three substrates that have been proposed as molecules transferred from vegetative cells to heterocysts are glutamate, sucrose, and alanine [2,4,5,6,7]. Sucrose is a universal vehicle of reduced carbon in plants [8], and it seems to be important also in cyanobacteria [9]; alanine can be an immediate source of reducing power in the heterocyst, where it is metabolized by alanine dehydrogenase [6]; and glutamate is mainly synthesized in vegetative cells by glutamate synthase and used in the heterocysts by glutamine synthetase to produce glutamine [2,10]. Heterocysts accumulate cyanophycin, a non-ribosomically synthesized peptide made of aspartate and arginine (multi-l-arginyl-poly [l-aspartic acid]) [11]. Cyanophycinase produces β-aspartyl-arginine, which is hydrolyzed by isoaspartyl dipeptidase in the vegetative cells [3]. Thus, glutamine and β-aspartyl-arginine (and perhaps also aspartate and arginine) appear to be nitrogen vehicles from heterocysts to vegetative cells [3,12].

Two possible pathways for intercellular molecular exchange in heterocyst-forming cyanobacteria have been discussed [1,13,14,15]. One involves structures at the septum between cells, septal junctions, which may consist of protein complexes that traverse septal peptidoglycan [16]. Septal junctions appear to allow the exchange of small, water-soluble compounds between cells [17,18]. Possible components of these cell–cell connecting structures are the SepJ, FraC, and FraD proteins [19,20]. The other route for cell–cell communication is the continuous periplasm present in the cyanobacterial filament [13,21]. The exchange of a substance through this route would require export to the periplasm in the producing cell, diffusion through this compartment, and import into the recipient cell. Interestingly, the outer membrane, which is continuous along the filament, appears to be relatively impermeable for compounds such as sucrose and glutamate that are exchanged between cells [22]. Independently of the pathway that might be used for nutrient exchange, cytoplasmic membrane transporters appear to be important in the diazotrophic physiology of heterocyst-forming cyanobacteria, since some amino acid transport mutants of the model heterocyst-forming cyanobacterium *Anabaena* sp. strain PCC 7120 (hereafter *Anabaena*) are impaired in diazotrophic growth [23].

*Anabaena* is able to take up from the external medium a broad range of amino acids independently of whether it has been grown with combined nitrogen or under diazotrophic conditions [23,24,25,26,27,28]. This ability has been ascribed to the activity of several transport systems, termed N-I, N-II, and Bgt, which show different specificities for amino acids [27,28]. N-I recognizes 20 proteinogenic amino acids except for aspartate and is the main transporter responsible for the uptake of hydrophobic amino acids, especially proline, although it also transports some other amino acids including glutamine and glutamate. N-II recognizes and transports mainly acidic (aspartate and glutamate) and neutral polar amino acids, again including glutamine. Finally, the Bgt system is a basic amino acid transporter that also contributes to the uptake of glutamine, an amino acid that can therefore be transported by the three identified transporters of *Anabaena*. These three systems are ABC-type uptake transporters, and their constituents are summarized in Table 1. Of note, the *bgtA* gene encodes an ATP-binding subunit that energizes transport by the N-II and Bgt systems [28].

**Table 1:** ABC-type amino acid uptake transporters of *Anabaena* sp. strain PCC 7120. The Transporter Classification Database (TCDB) family number [29,30] is indicated. The order in which the transported amino acids are presented for each transporter reflects the contribution of the corresponding transporter to the total uptake of the indicated amino acids by nitrate-grown *Anabaena* filaments; bold face, preferred amino acids. ATPase: ATP-binding and hydrolyzing protein; Orn: ornithine; PSB: periplasmic substrate-binding protein; TM: transmembrane (permease) protein. Cys, Trp, and Tyr have not been investigated in transport assays in *Anabaena*.

Transporter	TCDB #	Transported amino acids	ORF	Gene	Predicted gene product	Ref.
N-I	3.A.1.4	**Pro**, **Phe**, **Leu**,	*all1046*	*natA*	ATPase	[27]
		Gly, Thr, Ala,	*alr1834*	*natB*	PSB	
		Ser, Met, Asn,	*all1047*	*natC*	TM	
		His, Orn, Gln,	*all1248*	*natD*	TM	
		Glu	*all2912*	*natE*	ATPase	
N-II	3.A.1.3	**Asp**, Glu, Asn,	*alr4164*	*natF*	PSB	[28]
		Gln, Met, Thr,	*alr4165*	*natG*	TM	
		Ala, Ser, Gly,	*alr4166*	*natH*	TM	
		His	*alr4167*	*bgtA*	ATPase	
Bgt	3.A.1.3	**Lys**, **Arg**, Orn,	*alr4167*	*bgtA*	ATPase	[28]
		His, Gln	*alr3187*	*bgtB*	PSB and TM	
N-III	3.A.1.4	**Gly**, Pro, Glu,	*alr2535*	*natI*	PSB	This work
		Phe, Leu, Ala,	*alr2536*	*natJ*	TM	
		Gln	*alr2538*	*natK*	TM	
			*alr2539*	*natL*	ATPase	
			*alr2541*	*natM*	ATPase	

Some cyanobacterial mutants have been reported to accumulate amino acids in the culture medium [6,23,27,28,31,32]. In *Anabaena*, release of hydrophobic amino acids is a general feature of strains mutated in neutral amino acid transporters [23,27,28]. This is especially relevant under diazotrophic conditions, where alanine released from strains mutated in the N-I and N-II systems reaches a concentration of 0.1 mM in the medium [28].

In this work we have investigated additional Open Reading Frames (ORF) of the *Anabaena* genome encoding proteins that show homology with amino acid transporters. We generated inactivation mutants of these ORFs and studied their amino acid uptake activity. Moreover, with the aim of clarifying aspects of the loss of amino acids to the external medium, we set up an assay to quantify the release from the cells of an alanine analogue that cannot be metabolized.

## 2. Experimental Section 

### 2.1. Bacterial Strains and Growth Conditions

*Anabaena* sp. (also known as *Nostoc* sp.) strain PCC 7120 was grown in BG11 (which contains NaNO_3_) [33] or BG11_0_ (free of combined nitrogen) medium at 30 °C in the light (25–75 μmol m^−2^ s^−1^), in shaken (80–90 rpm) liquid cultures or in medium solidified with 1% Difco agar. When indicated, the medium was supplemented with 10 mM NaHCO_3_ and the cultures were bubbled with 1% CO_2_ (BG11C or BG11_0_C). For the mutants described below, antibiotics were used at the following concentrations: streptomycin sulfate (Sm), 2–5 μg mL^−1^; spectinomycin dihydrochloride pentahydrate (Sp), 2–5 μg mL^−1^ and neomycin sulfate (Nm), 5–30 μg mL^−1^ for liquid cultures; and Sm, 5 μg mL^−1^; Sp, 5 μg mL^−1^ and Nm, 40 μg mL^−1^ for solid cultures. DNA from *Anabaena* sp. was isolated by the method of Cai and Wolk [34].

*Escherichia coli* DH5α was used for plasmid constructions. This strain and strains HB101 and ED8654, used for conjugation to *Anabaena*, were grown in LB medium, supplemented when appropriate with antibiotics at standard concentrations [35].

### 2.2. Plasmid Construction and Genetic Procedures

For inactivation of *alr2536*, *all0342*, and *alr3429*, internal fragments of 552 bp, 596 bp, and 469 bp, respectively, were amplified by PCR using DNA from strain PCC 7120 as a template and primers alr2536-7120-1 and alr2536-7120-2 for *alr2536*, all0342-7120-1 and all0342-7120-2 for *all0342*, and alr3429-7120-1 and alr3429-7120-2 for *alr3429* (all primers contain *Bam*HI restriction sites in their 5' ends and are listed in Table 2). Amplified fragments were cloned into vector pGEM-T (Promega) in the case of *alr2536* and *all0342* or pGEM-T Easy (Promega) for *alr3429*, producing pCSR4, pCSR1, and pCSR19, respectively, and then transferred as *Bam*HI-ended fragments to *Bam*HI-digested pRL424 [36] producing pCSR17, pCSR13, and pCSR23, respectively (Nm^r^).

For inactivation of *alr1519*, a 2.3 kb DNA fragment carrying the full ORF and flanking regions was amplified from genomic DNA from strain PCC 7120 using primers AA-1 and AA-2. The amplified fragment was inserted into vector pGEM-T (Promega) producing pCSS1. The C.S3 cassette (encoding Sm^r^ Sp^r^) was excised from plasmid pRL463 [37] with *Bam*HI and inserted into the *Bcl*I-digested pCSS1 to interrupt the ORF *alr1519*, obtaining plasmid pCSS2. The construct was transferred as a *Pvu*II-ended fragment to the suicide plasmid pRL278 [38] digested with *Nru*I producing pCSS4 (Sm^r^ Sp^r^ Nm^r^).

For inactivation of *all1189, alr1538*, and *all3551*, internal fragments of 439 bp, 440 bp, and 417 bp, respectively, were amplified by PCR using DNA from strain PCC 7120 as a template and primers all1189-1 and all1189-2 for *all1189*, alr1538-1 and alr1538-2 for *alr1538*, and all3551-1 and all3551-2 for *all3551* (all primers contain *Bam*HI restriction sites in, or close to, their 5' ends). Amplified fragments were cloned into vector pMBL-T (MBL) producing pCSVM1, pCSVM2, and pCSVM3, respectively, and then transferred as *BamH*I-ended fragments to *BamH*I-digested pCSV3 producing, respectively, pCSVM4, pCSVM5, and pCSVM6 (Sm^r^ Sp^r^).

**Table 2:** Oligodeoxynucleotide primers used in this work. Introduced restriction enzyme cutting sites are indicated in boldface.

Primer	Sequence (5'→3')
alr2536-7120-1	**GGA TCC** GCT AAC GCT ACT TTG CCG
alr2536-7120-2	**GGA TCC** GCA ACC CAA AGC CAA TC
all0342-7120-1	**GGA TCC** GTT GAC CAA TAC CCT CAT GGC
all0342-7120-2	**GGA TCC** GCT TGG AAG GTT ACA GGC
alr3429-7120-1	**GGA TCC** GGG GTT TAA AGA TGC TGA CGG
alr3429-7120-2	**GGA TCC** GAG GAT GTT CTC TCA CCC
all1189-1	**GGA TCC** GGA AAC TCA CAG
all1189-2	GC**G GAT CC**A GGA TAA TAG
alr1538-1	**GGA TCC** TGG CTG TGT ATT TAG
alr1538-2	**GGA TCC** TTT GGG CAG AAG
all3551-1	**GGA TCC** AGC CCA ATA GTT G
all3551-2	**GGA TCC** CTG CCA AAG AC
AA-1	GAG CCA TAC AAG CTC TGA TTC ATG G
AA-2	ACG CGA TCG CTG ACT CCT GCC

Conjugation of *Anabaena* sp. strain PCC 7120 with *E. coli* HB101 carrying plasmids pCSR17, pCSR13, pCSR23, pCSS4, pCSVM4, pCSVM5, or pCSVM6 with helper and methylation plasmid pRL623 was effected by the conjugative plasmid pRL443, carried in *E. coli* ED8654, and performed as described previously [39]. Exconjugants were selected for their resistance to Sm and Sp, or to Nm; for the generation of the mutant CSS4, double recombinants were selected for their resistance to sucrose. The genetic structure and segregation of selected clones were studied by Southern analysis using as probes the cloned fragments from the corresponding mutated gene (see above). The probes were labeled with Ready-To-Go DNA Labeling Beads (−dCTP) kit (GE Healthcare). The mutants, which were homozygous for the mutated chromosomes in all cases, were named as follows: CSR6 from plasmid pCSR17, CSR1 from plasmid pCSR13, CSR3 from plasmid pCSR23, CSS4 from plasmid pCSS4, CSVM1 from plasmid pCSVM4, CSVM2 from plasmid pCSVM5, and CSVM3 from plasmid pCSVM6.

### 2.3. Growth Tests and Sample Preparation

Growth tests were carried out by spotting 2–7 µL of cell suspension containing 2.5–10 µg Chl mL^−1^ on agar plates of BG11 or BG11_0_ medium. The plates were incubated at 30 °C in the light (25–40 µmol m^−2^ s^−1^) and observed over a period of two weeks. Growth rates in liquid medium were determined as previously described [23]. The growth rate constant (µ = ln2/*t_d_*, where *t_d_* is the doubling time) was calculated from the increase of protein content determined in 0.2 mL of samples of shaken liquid cultures. Protein concentration was determined by a modified Lowry procedure [40]. Chlorophyll *a* (Chl) content of cultures was determined by the method of Mackinney [41].

### 2.4. Substrate Transport Assays 

Amino acid uptake assays were carried out at 30 °C in the light (175 μmol m^−2^ s^−1^). Amino acid uptake was determined as described previously [23] in 10-min transport assays with the indicated concentration of l-[U-^14^C]amino acid, in filaments grown in BG11 medium (supplemented with the appropriate antibiotic in the case of the mutants), washed and suspended in 25 mM N-tris(hydroxymethyl)-methylglycine (Tricine)-NaOH buffer (pH 8.1). Significance of the difference of uptake between a mutant and the wild type was assessed by unpaired Student’s *t* tests assuming a normal distribution of the data. Differences with *P* ≤ 0.05 are considered statistically significant.

For solute release experiments, filaments grown in BG11 or BG11_0_ medium were washed, suspended in 25 mM Tricine-NaOH buffer (pH 8.1), and incubated with 3 μM α-[1-^14^C]aminoisobutyric acid (AIB) for 40 or 90 min at 30 °C in the light (175 μmol m^−2^ s^−1^). At the time indicated, an excess of unlabeled substrate at a concentration of 1 mM was added. Samples were removed at different times after the addition of the unlabeled substrate and cell-associated radioactivity was determined after filtration and washing with about 3–5 mL of 5 mM Tricine-NaOH buffer (pH 8.1).

To determine metabolites produced from the labeled substrate and released from the cells at the end of the experiments, samples of 0.2 mL of the cell suspension were centrifuged at 14,000× *g* for 1 min and supernatants were centrifuged again. Samples of the resulting solutions were applied to 0.1-mm-thick cellulose thin-layer chromatography (TLC) plates (20 cm × 20 cm; Merck, Darmstadt, Germany). Two-dimensional separation of amino acids was effected by using the following solvents: the first dimension solvent consisted of *n*-butanol-acetone-ammonium hydroxide-water (20:20:10:4, v/v/v/v), and the second dimension solvent consisted of isopropanol-formic acid-water (20:1:5, v/v/v). TLC plates were analyzed by electronic autoradiography using a two-dimensional scanner for β particles (Cyclone Plus Phosphor Imager, PerkinElmer, Waltham, MA, USA). 

## 3. Results

### 3.1. Predicted ABC-Type Amino Acid Transporters

**Figure 1:**
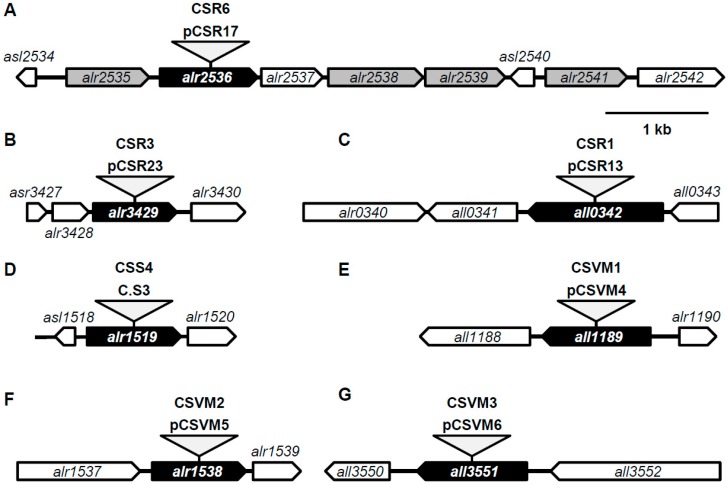
Insertional inactivation of ORFs *alr2536*, *alr3429*, *all0342*, *alr1519*, *all1189*, *alr1538*, and *all3551*. Schematic representation of the loci of *alr2536* (**A**); *alr3429* (**B**); *all0342* (**C**); *alr1519* (**D**); *all1189* (**E**); *alr1538* (**F**); and *all3551* (**G**), with indication of the plasmids inserted to produce strains CSR6, CSR3, CSR1, CSS4, CSVM1, CSVM2, and CSVM3, respectively.

Blast searches of the *Anabaena* genome, using as queries the amino acid sequences of the permease subunits of the *Anabaena* ABC-type transporters N-I and N-II (Table 1), identified a new cluster of genes (*alr2535* to *alr2541*) putatively encoding the elements of an additional ABC-type transporter for amino acids (Figure 1A). ORF *alr2535* would encode a protein of 268 amino acid residues with homology to the periplasmic substrate-binding protein BraC of the branched-chain amino acid transport system of *Pseudomonas aeruginosa* [42]*.* ORFs *alr2536* and *alr2538* would encode 316 and 308 amino acid residue peptides, respectively, with homology to permeases NatC and NatD of the transport system N-I of *Anabaena*. ORF *alr2539* and *alr2541* would encode 259 and 264 amino acid residue peptides, respectively, showing homology with ATPases NatA and NatE of the N-I system. Two additional ORFs, *alr2537* and *asl2540*, would encode hypothetical proteins with no homologues in the databases. 

To inactivate this putative ABC-type transporter, plasmid pCSR17, based on pRL424, which carries the *npt* gene with no transcriptional terminators [36], was inserted through a single cross-over event into *alr2536*, producing the *Anabaena* mutant strain CSR6 (Figure 1A). Strain CSR6 grew well on solid medium with or without combined nitrogen (not shown). The uptake of some amino acids was studied in nitrate-grown filaments of strain CSR6 in comparison to the wild type. The mutant was mainly impaired in the transport of hydrophobic amino acids (54%–72% of wild-type activity), especially glycine, and impairment in the transport of glutamate and glutamine (63% and 77% of wild-type activity, respectively) was also observed (Table 3). These results suggest that some of the genes in the *alr2535* to *alr2541* gene cluster encode components of a transport system that can mediate the uptake of some amino acids in *Anabaena*, mainly hydrophobic amino acids. We have named this system N-III, and the corresponding genes *natI* to *natM* (Table 1). 

The search for genes encoding other putative components of ABC-type amino acid transport systems identified ORF *alr3429*, which is annotated as GlnH, a periplasmic glutamine-binding protein [43], but is most similar to *Synechocystis* GtrC, a periplasmic glutamate-binding protein [44]. Although genes encoding an ABC transporter are frequently clustered together, *alr3429* upstream and downstream genes have no obvious relation to transport functions. ORF *alr3429* was inactivated by insertion of plasmid pCSR23 (based on pRL424) through a single cross-over event producing the *Anabaena* mutant strain CSR3 (Figure 1B). Strain CSR3 grew well on a solid medium with or without combined nitrogen (not shown). The uptake rate of representative amino acids in this mutant was analyzed, in comparison with the wild type, in filaments that had been grown with nitrate as the nitrogen source. For the tested amino acids, the activity was within a range of about ±20% the wild-type activity (Table 3), giving no support for a specific role of Alr3429 in amino acid transport.

**Table 3:** Amino acid transport activities in *Anabaena* sp. strain PCC 7120 and mutant strains. BG11-grown cells were used in 10-min transport assays with 10 μM [^14^C]amino acid substrate as described in the Experimental Section. Activities are given in nmol (mg Chl)^−1^ per 10 min; the mean ± standard deviation of the mean (SE) from the number of measurements indicated in parenthesis is presented. The percentage of the wild-type activity is also given for the mutants. Differences between mutant and wild-type activities were assessed by the Student’s *t* test (*P* indicated in each case).

Substrate	Transport activity (nmol [mg Chl]^−1^)						
	PCC 7120	Mutant strain (inactivated ORF)					
		CSR6 (*alr2536*)		CSR3 (*alr3429*)		CSR1 (*all0342*)	
	Mean ± SE (n)	Mean ± SE (n)	% (*P*)	Mean ± SE (n)	% (*P*)	Mean ± SE (n)	% (*P*)
***Basic***							
l-Arg	125 ± 1.59 (25)	115 ± 16.4 (3)	92% (0.077)	148 ± 6.60 (4)	119% (0.187)	195 ± 12.5 (3)	156% (0.068)
l-Lys	138 ± 1.55 (16)	151 ± 3.92 (2)	110% (0.167)	163 ± 6.15 (3)	118% (0.119)	140 ± 8.40 (2)	102% (0.882)
l-His	88.0 ± 1.10 (16)	71.7 ± 4.80 (3)	81% (0.174)	90.0 ± 4.94 (4)	102% (0.863)	132 ± 5.28 (3)	150% (0.023)
***Acidic***							
l-Asp	37.3 ± 0.39 (27)	33.5 ± 2.18 (3)	90% (0.440)	31.1 ± 1.67 (4)	83% (0.170)	38.8 ± 2.19 (4)	104% (0.768)
l-Glu	10.5 ± 0.12 (25)	6.62 ± 0.70 (3)	63% (0.061)	10.2 ± 0.26 (4)	97% (0.721)	16.9 ± 1.56 (4)	161% (0.130)
***Neutral polar***							
l-Gln	99.4 ± 0.90 (30)	77.0 ± 6.89 (3)	77% (0.189)	99.3 ± 6.31 (4)	100% (0.994)	158 ± 6.48 (4)	158% (0.014)
l-Ser	218 ± 2.50 (10)	195 ± 2.05 (2)	90% (0.022)	191 ± 9.37 (3)	88% (0.237)	239 ± 34.3 (3)	110% (0.753)
***Hydrophobic***							
l-Ala	192 ± 1.09 (20)	139 ± 13.4 (3)	72% (0.145)	203 ± 16.3 (4)	105% (0.772)	286 ± 6.03 (3)	149% (0.004)
Gly	200 ± 3.30 (15)	107 ± 14.0 (3)	54% (0.039)	222 ± 8.84 (4)	111% (0.337)	252 ± 5.50 (3)	126% (0.007)
l-Leu	103 ± 0.99 (17)	70.4 ± 5.34 (3)	68% (0.051)	117 ± 5.33 (4)	113% (0.315)	177 ± 12.0 (3)	171% (0.067)
l-Pro	135 ± 1.99 (16)	82.8 ± 14.9 (3)	61% (0.174)	151 ± 10.0 (3)	112% (0.449)	198 ± 13.1 (4)	147% (0.089)
l-Phe	118 ± 1.87 (16)	79.4 ± 1.78 (2)	67% (<0.001)	91.6 ± 7.79 (4)	78% (0.197)	109 ± 8.32 (3)	92% (0.607)

### 3.2. Predicted Amino Acid Transporters from Other Transporter Families

In the genome of *Anabaena,* ORF *all0342* encodes a predicted 437 amino acid peptide, annotated as H^+^/Na^+^-dependent glutamate and aspartate symporter [45], which belongs to the dicarboxylate/amino acid:cation symporter (DAACS) transporter family (TCDB family 2.A.23 [30]). ORF *all0342* was inactivated by insertion through a single cross-over event of pCSR13 (based on pRL424), producing *Anabaena* mutant strain CSR1 (Figure 1C). Strain CSR1 grew well on a solid medium with or without combined nitrogen (not shown). Regarding amino acid uptake, strain CSR1 showed about a 50% increase compared to the wild type in the uptake of some amino acids from all the chemical groups tested (Table 3). This increase was statistically significant for histidine, glutamine, alanine, and glycine (*P* ≤ 0.05). These results suggest either that All0342 participates in amino acid export, its mutation resulting in retention of some amino acids in the cells, or that lack of All0342 facilitates uptake mediated by other transporters. 

ORF *alr1519* encodes a predicted 456 amino acid peptide showing homology to transporters of the amino acid-polyamine-organocation (APC) family (TCDB 2.A.3 [30]) that includes solute:cation symporters and solute:solute antiporters [46,47]. ORF *alr1519* was inactivated by insertion of the C.S3 cassette (Sm^r^ Sp^r^ [36]), producing the mutant strain CSS4 (Figure 1D). The growth rate of CSS4 mutant in liquid BG11 medium was similar to that of the wild type (µ, 0.65 day^−1^ for both strains). However, under diazotrophic conditions the growth rate of the mutant was about 75% that of the wild type (0.36 ± 0.04 day^−1^ for the CSS4 mutant *versus* 0.43 ± 0.04 day^−1^ for the wild type; *n* = 3). Amino acid uptake was then tested in filaments of strain CSS4 and wild-type *Anabaena* grown in the presence and absence of combined nitrogen (Table 4). We found only a slight increase in the transport of aspartate, but this alteration was hardly statistically significant. These results give no support for a specific role of Alr1519 in amino acid uptake.

**Table 4:** Amino acid transport activities in *Anabaena* sp. strains PCC 7120 and CSS4. BG11C and BG11_0_C-grown filaments were used in 10-min transport assays with 10 μM [^14^C]amino acid substrate as described in the Experimental Section. Activities are presented in nmol (mg Chl)^-1^ per 10 min; the mean ± standard deviation of the mean (SE) from the number of measurements indicated in parenthesis is presented. The percentage of the wild-type activities is also given for the mutant. Differences between the mutant and wild-type activities were assessed by the Student’s *t* test (*P* indicated in each case).

Substrate	Transport activity (nmol [mg Chl]^−1^)					
	BG11C			BG11_0_C		
	PCC 7120	CSS4 (*alr1519*)		PCC 7120	CSS4 (*alr1519*)	
	Mean ± SE (n)	Mean ± SE (n)	% (*P*)	Mean ± SE (n)	Mean ± SE (n)	% (*P*)
***Basic***						
l-Arg	116 ± 7.64 (3)	113 ± 3.16 (2)	98% (0.864)	187 ± 8.96 (3)	167 ± 3.58 (2)	89% (0.327)
***Acidic***						
l-Asp	30.3 ± 1.03 (4)	42.0 ± 3.85 (2)	139% (0.214)	62.1 ± 0.98 (4)	77.6 ± 3.57 (3)	125% (0.119)
l-Glu	10.4 ± 1.48 (4)	10.3 ± 0.64 (3)	99% (0.970)	34.7 ± 1.63 (4)	32.2 ± 1.90 (3)	93% (0.619)
***Neutral polar***						
l-Gln	118 ± 4.46 (3)	125 ± 2.15 (2)	106% (0.487)	216 ± 5.30 (3)	214 ± 2.06 (2)	99% (0.854)
***Hydrophobic***						
l-Ala	224 ± 12.6 (3)	215 ± 17.8 (2)	96% (0.826)	309 ± 11.7 (3)	299 ± 14.0 (2)	108% (0.748)

### 3.3. Release of Amino Acids from Vegetative Cells

*Anabaena* mutants impaired in transport systems N-I and N-II release hydrophobic amino acids, especially alanine, to the culture medium [27,28]. To gain a better understanding of the kinetics of alanine release, the alanine analogue α-aminoisobutyric acid (AIB), which cannot generally be metabolized, was used. In the N-I mutant strain CSR11 (*natD*) [27], the rate of [^14^C]AIB uptake was 2.43 ± 0.48% (*n* = 3) that of the wild type, indicating that the N-I system is responsible for about 98% of the uptake of this compound in *Anabaena*.

**Figure 2:**
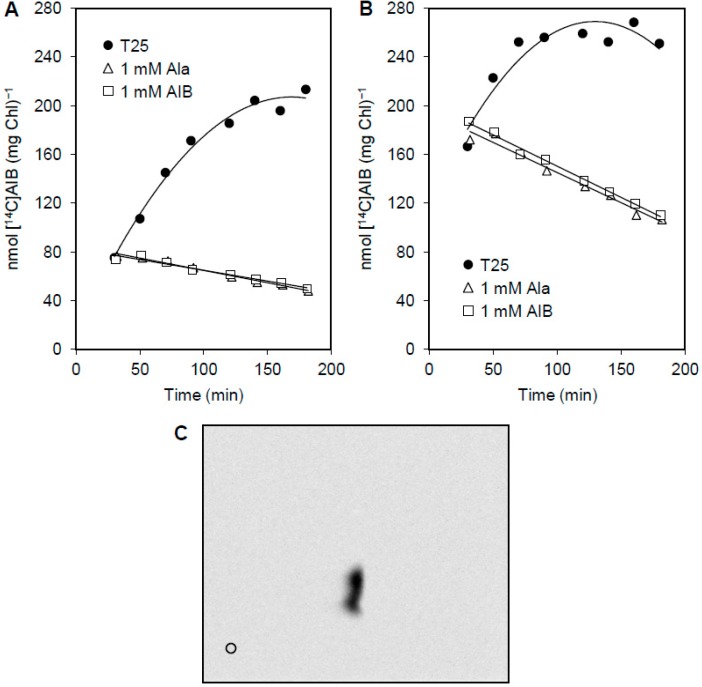
AIB release in *Anabaena*. Filaments from BG11 medium (**A**) or incubated in BG11_0_ medium for 24 h (**B**) were incubated in Tricine buffer with 3 μM [^14^C]AIB. After 40 min the filament suspensions were supplemented with 1 mM AIB or alanine in a small volume of buffer or, as a control, with the same volume of buffer (T25). Cell-associated ^14^C was measured in samples taken from the filament suspensions at the times indicated. (**C**) A sample of the medium from the end of the assay with BG11-grown cells incubated with [^14^C]AIB and supplemented with unlabeled AIB was subjected to TLC analysis; o: origin of the chromatography. Only one radioactive spot co-migrating with AIB was detected.

To assay the release of AIB in *Anabaena*, the following experiment was set up. Filaments grown in the presence of nitrate were incubated with 3 μM [^14^C]AIB for 40 to 90 min under transport assay conditions. Then, an unlabeled substrate was added at 1 mM concentration and the amount of [^14^C]AIB remaining in the cells was determined at different times. The release of [^14^C]AIB in response to the addition of unlabeled substrates could thus be quantified as the decrease in the amount of radioactivity remaining in the cells. In wild-type *Anabaena* grown with combined nitrogen and incubated with or without combined nitrogen for 24 h, significant release of [^14^C]AIB was observed in samples supplemented with unlabeled AIB or alanine, but not with a buffer (Figure 2A, B). Note that, at the concentrations used, [^14^C]AIB uptake likely became negligible when a competitor such as unlabeled AIB or alanine was added, but evidently not when only buffer (T25) was added. The ^14^C-labeled compound(s) released to the medium were analyzed by TLC and only one spot corresponding to AIB was detected (Figure 2C), consistent with the idea that AIB is not metabolized.

**Figure 3:**
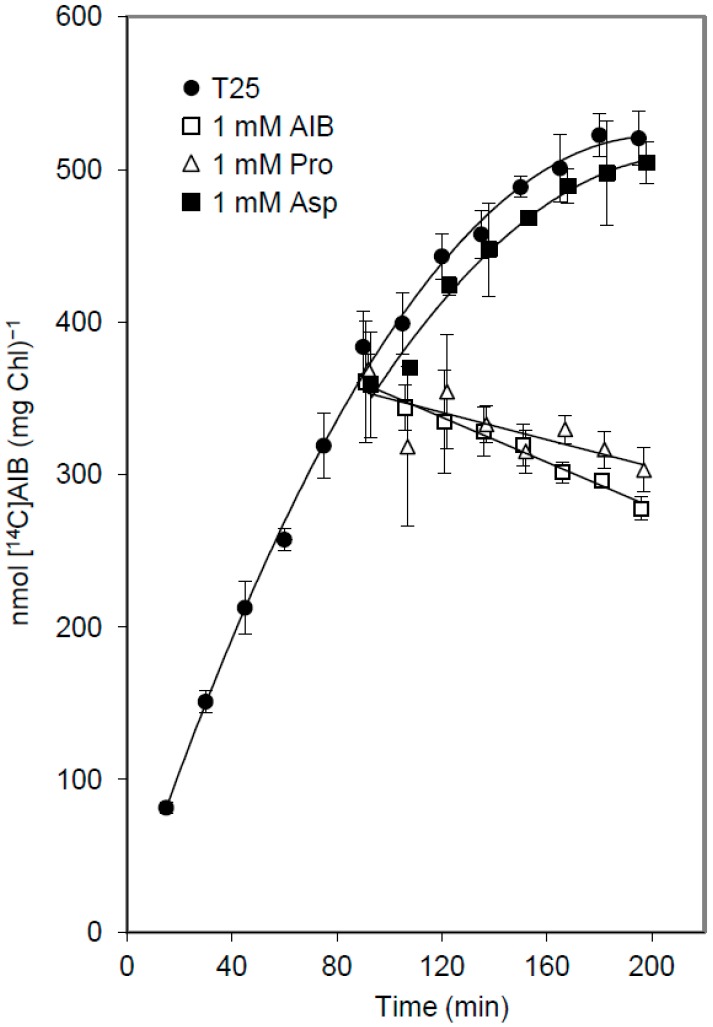
Specificity of AIB release in *Anabaena*. Filaments grown in BG11 medium were incubated in Tricine buffer with 3 μM [^14^C]AIB. After 90 min the filament suspensions were supplemented with 1 mM AIB, proline, or aspartate in a small volume of buffer or, as a control, with the same volume of buffer (T25). Cell-associated ^14^C was measured in samples taken from the filament suspensions at the times indicated.

The release of [^14^C]AIB in response to the addition of different concentrations of AIB ranging from 0.1 to 3 mM was assayed with no significant differences in the rate of release (not shown). To test whether the transport system N-I or N-II is involved in the release of [^14^C]AIB, assays were performed with specific substrates of these transporters. Filaments were incubated in the presence of 3 μM [^14^C]AIB for 90 min, and the release of [^14^C]AIB in response to the addition of an excess of unlabeled AIB, proline (a specific substrate of system N-I), or aspartate (a specific substrate of system N-II) was determined (Figure 3). Whereas the addition of proline produced the release of [^14^C]AIB in a similar way to AIB, the addition of aspartate did not affect [^14^C]AIB uptake, which continued in the presence of aspartate. These results indicate that net [^14^C]AIB release is dependent on the addition of a substrate of transport system N-I. We suggest that [^14^C]AIB, accumulated within the cells, is released to some extent and then re-incorporated through the N-I transporter, and that saturation of this transporter by unlabeled AIB, alanine (Figure 2), or proline (Figure 3) impedes re-incorporation, permitting us to observe net release.

**Figure 4:**
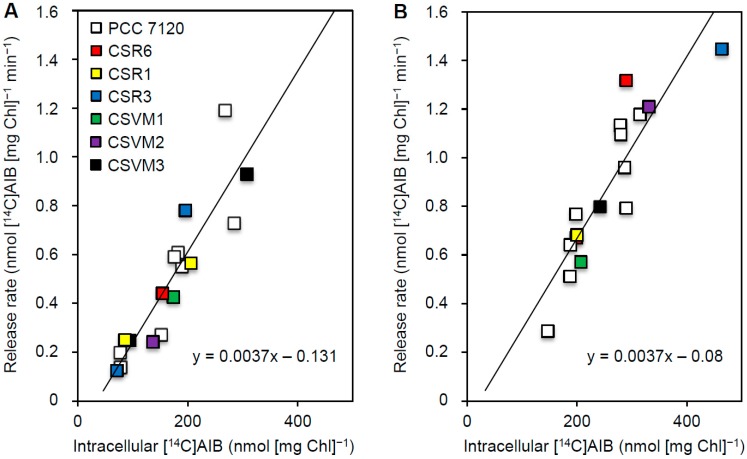
Kinetics of AIB release. The rate of [^14^C]AIB release in response to the addition of 1 mM unlabeled AIB to filaments incubated in Tricine buffer with 3 μM [^14^C]AIB was measured. Unlabeled AIB was added after 40 to 90 min of incubation with [^14^C]AIB. (**A**) Nitrate-grown filaments. (**B**) Filaments incubated in BG11_0_ medium for 24 h. Assuming an intracellular volume of 125 µL (mg Chl)^−1^, the range of intracellular [^14^C]AIB concentrations reached in the assays was from about 0.5 to 4 mM.

In the experiments of AIB release, we observed that the rate of release depended on the intracellular concentration of [^14^C]AIB reached in each particular cell suspension, which depended on the time at which unlabeled AIB was added (cell suspensions were assayed at the same growth stage in all experiments). Because the level of accumulated [^14^C]AIB was known for each assay, we could represent the rate of release as a function of the intracellular [^14^C]AIB concentration (see data for the wild type, PCC 7120, in Figure 4). A linear relation, *y* = 0.004*x* + *b*, where *y* is the release rate and *x* the intracellular concentration of [^14^C]AIB, could be established, with r^2^ coefficients of 0.779 for BG11-grown filaments and 0.913 for filaments that had been incubated in the absence of combined nitrogen (BG11_0_ medium).

Blast searches of the *Anabaena* genome for genes homologous to exporters from the EamA (amino acid, metabolite efflux pumps) family (TCDB 2.A.7.3.2, which belongs to the Drug and Metabolite Exporter [DME] family, TCDB 2.A.7.3 [30]) identified three open reading frames: *all1189, alr1538*, and *all3551*. Each of these ORFs was inactivated by insertion through single cross-over of a plasmid based on pCSV3 (Sm^r^ Sp^r^). Insertion of pCSVM4, pCSVM5, and pCSVM6 produced mutant strains CSVM1, CSVM2, and CSVM3, respectively (Figure 1). The three mutants grew similarly to the wild type both in the presence and in the absence of combined nitrogen (not shown). Activity of [^14^C]AIB release in response to the addition of unlabeled AIB was tested in mutant strains CSVM1, CSVM2, and CSVM3, as well as in CSR1, CSR6, and CSR3 described above. Significant export was observed in every case, providing no indication of the involvement in AIB release of the protein encoded by any of the mutated genes. Instead, we observed that the rate of [^14^C]AIB release in the mutants fitted the relation to intracellular [^14^C]AIB concentration previously established for the wild type. Adding the data from the mutants to those of the wild type, the same linear relation was observed with r^2^ coefficients of 0.818 for BG11-grown filaments and 0.764 for filaments that had been incubated in BG11_0_ medium. The linear dependence of the rate of AIB release on the intracellular concentration of AIB suggests that the release takes place by diffusion. Additionally, the fact that *b* takes a negative value (−0.131 for BG11-grown cells and −0.080 for BG11_0_-incubated cells) implies that there was an intracellular concentration threshold for the observation of [^14^C]AIB release. Assuming an intracellular volume of 125 µL (mg Chl)^−1^ [48,49], this threshold was in the range 0.17–0.28 mM. This concentration might correspond to AIB immobilized through its interaction with cellular components.

## 4. Discussion

In this work we have investigated new possible amino acid transporters encoded in the genome of the heterocyst-forming cyanobacterium *Anabaena* sp. strain PCC 7120. The amino acid transport systems previously known in this cyanobacterium are the ABC-type transporters N-I, N-II, and Bgt [23,27,28]. The genes encoding the elements of each of these transporters are summarized in Table 1. The N-I system is encoded by five genes, which are spread in the genome: *natA* (*all1046*) and *natE* (*all2912*) encode ATPases, *natC* (*all1047*) and *natD* (*all1284*) encode transmembrane (permease) proteins, and *natB* (*alr1834*) encodes a periplasmic substrate-binding protein [27]. The system N-II is encoded by a cluster of four genes: *natF* (*alr4164*) encodes a periplasmic substrate-binding protein, *natG* (*alr4165*) and *natH* (*alr4166*) encode transmembrane (permease) proteins, and *bgtA* (*alr4167*) encodes an ATPase [28]. The third one, the system Bgt, is the product of *bgtB* (*alr3187*), which encodes a permease protein with an extended periplasmic domain with homology to substrate binding proteins, and *bgtA* (*alr4167*), which encodes an ATPase that is shared with the system N-II [28]. 

Seven loci have been investigated here, but evidence for the involvement in amino acid transport was only found for two of them (see Results). A cluster of genes encoding the elements of a new ABC-type amino acid uptake transporter has been identified in this work: *alr2535* encodes a predicted periplasmic binding protein, *alr2536* and *alr2538* encode predicted transmembrane (permease) proteins, and *alr2539* and *alr2541* encode predicted ATPases. As judged from the transport activities displayed by an *alr2536* insertional mutant, this putative transporter can mediate uptake of glycine and other hydrophobic amino acids (Table 1). We have termed this amino acid transporter system N-III. Similarly to the previously characterized amino acid transport systems N-I and N-II of *Anabaena*, system N-III mainly transports one group of amino acids (hydrophobic amino acids), but has a relatively low specificity, being able to mediate as well the uptake of some other amino acids such as glutamate and glutamine. Nonetheless, system N-III is phylogenetically most similar to transporters in the Hydrophobic Amino Acid Uptake Transporter (HAAT) family (TCDB 3.A.1.4 [30]), as the N-I system is.

Members of the DAACS family of transporters (TCDB 2.A.23 [30]) are involved in the uptake of different substrates, including amino acids, mediated by a solute:Na^+^ symport mechanism [45]. *Anabaena* ORF *all0342* encodes a protein that is homologous to members of this family. The analysis of a mutant of this gene showed increased uptake of a number of amino acids. These results could be interpreted assuming that All0342 participates in amino acid export with a low specificity, since basic, neutral, and hydrophobic amino acids were affected. An effect on amino acid export implies that blocking export would result in increased retention within the cells of amino acids taken up by different transporters. Alternatively, All0342 might work in amino acid uptake competing with other transporters, so that the lack of All0342 would facilitate uptake mediated by those transporters.

Release of amino acids to the culture medium is a characteristic feature of some amino acid transport mutants. In the *Anabaena* *nat* mutants the set of released amino acids includes alanine, glycine, isoleucine, leucine, phenylalanine, proline, tyrosine, and valine, which are transported mainly by system N-I [23,27]. Because the released amino acids are all hydrophobic, they may leak out of the cells by diffusion, and a function of the N-I system may be the recapture of these leaked amino acids [23,31,32]. In order to test this further, a method to study kinetically the release of AIB from vegetative cells has been set up. Release of AIB is observed only when the N-I system is engaged in the uptake of specific substrates of this transporter, but not when a specific substrate of the N-II system is added. Because the N-I system is responsible for 98% of the uptake of AIB, these data support a function of the N-I system in recapturing the leaked amino acids. With the aim of identifying possible molecular actors of the release of AIB from *Anabaena* cells, the kinetics of AIB release was studied in wild-type *Anabaena* and some of the mutants generated in this work. The rate of release of AIB directly depends on the intracellular concentration of the substrate, following a first-order equation, which is consistent with diffusion. This could be simple diffusion through the lipid bilayer [50,51] or facilitated diffusion [52]. None of the investigated mutants was, however, impaired in release of AIB and, indeed, all of them showed the same trend as the wild type. Thus, if release were facilitated, proteins encoded by genes other than those inactivated in this work would be involved. We suggest that hydrophobic amino acids can leak out from the *Anabaena* filaments to the culture medium and that the ABC-type transporter N-I is important for recapturing the amino acids that have been lost from the cells. This function could be especially relevant under diazotrophic conditions, in which nitrogen is at a premium.

The genome of *Anabaena* is predicted to contain 6223 genes [53], of which 481 are predicted to encode transport proteins [54]. ABC-type transporters are particularly abundant in cyanobacteria as compared to other bacteria [55], and proteins constituting ABC-type transporters represent in *Anabaena* about 61% of the total number of membrane transporter proteins [54]. The ample use of ABC-type transporters in cyanobacteria may be related to the fact that in these organisms the bulk of energy conservation takes place in intracellular membranes, the photosynthetic thylakoids, which are separated from the cytoplasmic membrane. ATP generated in the thylakoids can then be used for cytoplasmic membrane transport directly by ABC-type transporters. Whereas many transporters in *Anabaena*, including ABC-type and other transporters, are involved in the uptake of inorganic nutrients [56], there are also many transporters that mediate the uptake of organic substances including, for instance, sugars [22,57] and oxo-acids [58]. The mainly photoautotrophic lifestyle of organisms such as *Anabaena* and most cyanobacteria may be therefore complemented by the ability to utilize organic compounds that could be found in their natural habitats.

## 5. Conclusions 

The genome of the heterocyst-forming cyanobacterium *Anabaena* sp. strain PCC 7120 contains a large number of genes encoding membrane transport proteins, many of which are elements of ABC-type transporters. Here we identified a new ABC-type uptake transporter, system N-III, mainly involved in the uptake of hydrophobic amino acids. We also identified a membrane protein, All0342, homologous to transporters of the dicarboxylate/amino acid:cation symporter (DAACS) family, whose mutation results in an increased accumulation of a wide range of amino acids in uptake assays. Finally, studying α-aminoisobutyric acid (AIB) export, we found that release of hydrophobic amino acids from cyanobacterial cells likely takes place by means of diffusion.
